# Building national patient registries in Mexico: insights from the MexOMICS Consortium

**DOI:** 10.3389/fdgth.2024.1344103

**Published:** 2024-06-04

**Authors:** Paula Reyes-Pérez, Ana Laura Hernández-Ledesma, Talía V. Román-López, Brisa García-Vilchis, Diego Ramírez-González, Alejandra Lázaro-Figueroa, Domingo Martinez, Victor Flores-Ocampo, Ian M. Espinosa-Méndez, Lizbet Tinajero-Nieto, Angélica Peña-Ayala, Eugenia Morelos-Figaredo, Carlos M. Guerra-Galicia, Estefania Torres-Valdez, María Vanessa Gordillo-Huerta, Nadia A Gandarilla-Martínez, Karla Salinas-Barboza, Guillermo Félix-Rodríguez, Gabriel Frontana-Vázquez, Yamil Matuk-Pérez, Ingrid Estrada-Bellmann, Deshiré Alpizar-Rodríguez, Mayela Rodríguez-Violante, Miguel E. Rentería, Alejandra E. Ruíz-Contreras, Sarael Alcauter, Alejandra Medina-Rivera

**Affiliations:** ^1^ Laboratorio Internacional de Investigación Sobre el Genoma Humano, Universidad Nacional Autónoma de México, Santiago de Querétaro, Mexico; ^2^ Departamento de Neurobiología Conductual y Cognitiva, Instituto de Neurobiología, Universidad Nacional Autónoma de México, Santiago de Querétaro, Mexico; ^3^ Laboratorio de Neurogenómica Cognitiva, Unidad de Investigación de Psicobiología y Neurociencias, Coordinación de Psicobiología y Neurociencias, Facultad de Psicología, Universidad Nacional Autónoma de México, Ciudad de México, Mexico; ^4^ Unidad de Genómica Avanzada, Langebio, Centro de Investigación y Estudios Avanzados del Instituto Politécnico Nacional, Irapuato, Mexico; ^5^ Escuela Nacional de Estudios Superiores, Unidad Juriquilla, Universidad Nacional Autónoma de México, Santiago de Querétaro, Mexico; ^6^ Hospital General Regional No. 1, Instituto Mexicano del Seguro Social, Querétaro, Santiago de Querétaro, Mexico; ^7^ Instituto Nacional de Rehabilitación “Luis Guillermo Ibarra Ibarra”, Ciudad de México, Mexico; ^8^ Hospital Regional, Instituto de Seguridad y Servicios Sociales de los Trabajadores del Estado, Morelia, Mexico; ^9^ Neurociencias PRISMA AC, San Luis Potosí, Mexico; ^10^ Hospital General Regional No. 2, Instituto Mexicano del Seguro Social, El Marqués, Mexico; ^11^ Hospital General Querétaro, Instituto de Seguridad y Servicios Sociales de los Trabajadores del Estado, Santiago de Querétaro, Mexico; ^12^ Departament of Neurology, Neurological Center, ABC Medical Center, México City, México; ^13^ Hospital General de México, Ciudad de México, Mexico; ^14^ Hospital Star Médica Querétaro, Santiago de Querétaro, Mexico; ^15^ Facultad de Medicina, Universidad Autónoma de Querétaro. Unidad de Neurociencias, Hospital Angeles Centro Sur, Santiago de Querétaro, Mexico; ^16^ Movement Disorders Clinic, Neurology Division, Internal Medicine Department, University Hospital “Dr. José E. González”, Universidad Autónoma de Nuevo León, Monterrey, Mexico; ^17^ Research Unit, Colegio Mexicano de Reumatología, Ciudad de México, Mexico; ^18^ Laboratorio Clínico de Enfermedades Neurodegenerativas, Instituto Nacional de Neurología y Neurocirugía Manuel Velasco Suárez, Mexico City, Mexico; ^19^ Mental Health and Neuroscience Program, QIMR Berghofer Medical Research Institute, Brisbane, QLD, Australia; ^20^ School of Biomedical Sciences, Faculty of Medicine, The University of Queensland, Brisbane, QLD, Australia

**Keywords:** patient registries, genetics, twins, systemic lupus erythematosus, Parkinson Disease

## Abstract

**Objective:**

To introduce MexOMICS, a Mexican Consortium focused on establishing electronic databases to collect, cross-reference, and share health-related and omics data on the Mexican population.

**Methods:**

Since 2019, the MexOMICS Consortium has established three electronic-based registries: the Mexican Twin Registry (TwinsMX), Mexican Lupus Registry (LupusRGMX), and the Mexican Parkinson's Research Network (MEX-PD), designed and implemented using the Research Electronic Data Capture web-based application. Participants were enrolled through voluntary participation and on-site engagement with medical specialists. We also acquired DNA samples and Magnetic Resonance Imaging scans in subsets of participants.

**Results:**

The registries have successfully enrolled a large number of participants from a variety of regions within Mexico: TwinsMX (*n* = 2,915), LupusRGMX (*n* = 1,761) and MEX-PD (*n* = 750). In addition to sociodemographic, psychosocial, and clinical data, MexOMICS has collected DNA samples to study the genetic biomarkers across the three registries. Cognitive function has been assessed with the Montreal Cognitive Assessment in a subset of 376 MEX-PD participants. Furthermore, a subset of 267 twins have participated in cognitive evaluations with the Creyos platform and in MRI sessions acquiring structural, functional, and spectroscopy brain imaging; comparable evaluations are planned for LupusRGMX and MEX-PD.

**Conclusions:**

The MexOMICS registries offer a valuable repository of information concerning the potential interplay of genetic and environmental factors in health conditions among the Mexican population.

## 1 Introduction

### 1.1 Brief history of patient registries

Patient registries are organized systems that collect, store, and analyze information about individuals from a specific population or living with a particular health condition (1). They are used for clinical research, recruitment into clinical trials, and improving patient care (2). When coupled with genomic information, they provide further opportunities for genomic medicine and research (3).

The National Leprosy Registry of Norway, established in 1856, was presumably the first national patient registry, implemented as a response to the high prevalence of leprosy at the time and included a collaborative effort between health officers and ministers of the church, which resulted in the policy of isolation that ultimately led to the disappearance of leprosy in Norway (4).

Since then, several registries have been set up around the globe with various aims. As expected, the two leading causes of death, cancer and heart disease, have led the efforts through the past century. There are at least 343 cancer registries in 65 countries (5), while 155 Cardiac disease registries in 49 countries have enrolled 73.1 million patients (6). One of the greatest examples is the US National Cardiovascular Data Registry (NCDR), which comprises ten registries and has more than 60 million records now entering the qualification of Big Data (7). In Latin America, population-based cancer registries, arguably the most extensively documented among patient registries, cover 20% of the population, and it is estimated that only 7% contain high-quality clinical information (8).

Although patient registries usually aim to estimate the prevalence of a disease or trait within a population and establish what demographics and environmental factors affect risk, they don't always aim to estimate how much genetic or environmental factors explain a trait. Historically, twin registries have been frequently used to improve our comprehension of human health and the genetic and environmental basis of complex diseases (9). The classical twin design consists of acquiring phenotypic data of monozygotic (MZ) and dizygotic (DZ) twins and measuring how correlated a trait is within pairs of both types. The heritability of a trait, the percentage of variance explained by genetic factors, can be estimated through the difference between the correlation of MZ twins and DZ twins, and the remaining variance in the trait is attributed to environmental factors (10). However, other family research designs can be used to estimate heritability. The so-called extended twin design acquires data from other family members of the twin participants, potentially improving the accuracy of heritability estimates (11). Genome-wide association studies (GWAS) can be used to estimate SNP-based heritability (12). Nevertheless, the costs of expanding twin studies or performing a GWAS are prohibitive for Lower and Middle-Income Countries that systematically lack funding for scientific research (13), making twin studies a more viable option.

Twin studies serve not only to estimate the heritability of a trait but also to assess how two different traits share genetic and environmental factors, elucidating their variance. Furthermore, they can assist in establishing causal relationships between associated traits (14). Consequently, insights gained from twin registries have deepened our understanding of genotype-environment interactions and their role in shaping the mechanisms underlying individual differences and their impact on health and disease conditions (9).

Despite these contributions, it is important to note that results from twin studies may not directly apply to populations with different genetic and environmental backgrounds. Latin America allegedly has some of the smallest sample sizes for twin registries, with around 5,000 individuals, Brazil has the most participants (*n* = 4,826), followed by Mexico (9).

The often mandatory implementation of patient records and twin registries across some countries has promoted relevant progress (15). Nevertheless, a considerable fraction of the world's population remains uncovered, particularly in African and Asian countries (16), due to the struggle to collect and integrate data to establish and maintain national registries (17).

### 1.2 Advancements and challenges in Mexican health registries

Although healthcare in Mexico has undoubtedly improved its coverage and scope over the last two decades (18), according to the 2020 census, about 33 million people in the country remain with no health services insurance (19), and 48.49% have no adequate access to health services (18).

Relevant efforts have been undertaken to establish registries in Mexico aimed at understanding the leading causes of death, which include heart disease, diabetes, and malignant tumors (20). These initiatives include the Mexican Registry of Atrial Fibrillation (ReMeFa) (21), the Mexican Registry of Pediatric Cardiac Surgery (22), and the National Registry of Cardiac Rehabilitation Programs (RENAPREC), alongside others focused on atrial fibrillation (23) and diabetes (24).

In some cases, efforts must be reinforced; for instance, the National Heart Failure Registry Program (25) was initiated in 2002 to generalize the characterization of patients with this condition; once the aim was achieved in 2004, the project was no longer pursued.

Although the government established a federal norm (NOM-024-SSA3-2012) in 2012 to regulate Electronic Health Record Information Systems (26), the diversity of electronic clinical records and database storage systems greatly hinders data sharing and interaction among health institutions (27). In addition, these registries are mainly survey-based and lack biobanking or genetic analyses, unlike their homologs in the European population, which limits their scope (28–30).

Biobanking encompasses the collection, preservation, analysis, and integration of biological information from biological samples along with personal and health-associated data (31). A few projects have been initiated in Mexico to advance biobanking efforts.

A partnership between the Carlos Slim Foundation and the Broad Institute launched the Slim Initiative in Genomic Medicine for the Americas (SIGMA), focusing on the study of type 2 diabetes (T2D), which has collected over 3,800 T2D cases and 4,072 healthy controls, of which 1,522 cases and 1,546 controls have been integrated into the 52K T2D exome sequencing study (32). The Metabolic Analysis in an Indigenous Sample (MAIS) cohort, which evaluated genetic contribution to traits associated with metabolic syndrome, has recovered samples of over 2,500 individuals from 60 different ethnic groups of Mexico (33).

The Mexico City Prospective Study (MCPS) and the Mexican Biobank (MXB) represent Mexico's most ambitious biobanking efforts. The MCPS aims to assess associations between risk factors and common causes of death in Mexico, including heart disease, stroke, diabetes, and alcoholic liver disease (34), and has generated genotyping and exome-sequencing for over 140,000 and whole genome sequencing of 9,950 individuals; this study is focused only on inhabitants from two districts (Coyoacán and Iztapalapa) in Mexico City (35).

Furthermore, MXB has analyzed over 6,000 DNA samples from over 800 rural and urban locations across Mexico and will serve as a reference for demographic and evolutionary studies. MXB includes anthropometric, disease, and lifestyle variables and biochemical traits derived from blood samples (36).

These two projects have generated invaluable genetic resources from Mexican individuals. However, they are constrained by limitations in the collected data and ethical restrictions when re-contacting participants. Specifically, there is minimal or no information regarding family history, mental and reproductive health, cognition, and chronic, degenerative, and immune diseases.

The MexOMICS Consortium was established to design and implement the required infrastructure for consolidating electronic-based databases and biobanks. This infrastructure is intended to collect, compare, cross-reference, and share valuable clinical, genetic, sociodemographic, and psychosocial information about Mexican individuals with various healthcare-related conditions. The objective is to facilitate a comprehensive characterization of our population.

The consortium is currently focused on three traits in the admixed Mexican population: (a) twins and members of multiple births (TwinsMX) (37), (b) people with Systemic Lupus Erythematosus (LupusRGMX) (38), and (c) patients with Parkinson Disease (MEX-PD) (39). There is a lack of other nationwide registries that include genetic data and publicly available information characterizing Mexican people with these traits. The developed databases offer valuable clinical and sociodemographic insights and include genetic profiling, biobanking, and the acquisition of Magnetic Resonance Imaging (MRI). These comprehensive efforts aim to deepen our understanding of genetic-environmental interactions and their impact on the phenotypic traits within the Mexican population, shedding light on potential implications for health and disease. In the present work, we will discuss the design, implementation, operational issues, and the current state and future directions of these three registries.

## 2 Methods

### 2.1 Ethics and data protection

The Research Ethics Committee of the Instituto de Neurobiología at the Universidad Nacional Autónoma de México (UNAM) approved all three registries. TwinsMX (40) was the first registry, established in 2019, followed by LupusRGMX and MEX-PD in 2021. Information is anonymized and stored at the Laboratorio Nacional de Visualización Científica Avanzada at UNAM. Participants provided informed consent before registration, and a privacy statement was given to them per the Federal Law on the Protection of Personal Data Held by Private Parties.

### 2.2 Platform and surveys design and implementation

Each registry follows specific guidelines to obtain necessary data. For such purposes, surveys are available on the web-based application Research Electronic Data Capture (REDCap), which allows secure data capture and storage (41, 42). Instruments integrated in the three registries are summarized in Table 1 to provide a general picture of the information being collected.

**Table 1:** Instruments employed and information required in twinsMX, LupusRGMX, and MEX-PD registries. The variables and instruments shared by two or more registries are in bold letters.

	Description		
Section	TwinsMX	LupusRGMX	MEX-PD
Sociodemographic variables	Full name Date of birth Age Contact information Occupation Last educational degree (participants and parents) State of residency Family's income in minimum wages	Full name Date of birth Age Contact information Occupation Last educational degree State of residency Number of habitants in home Socioeconomic level index rule of the Mexican Association of Market and Opinion Intelligence Agencies (AMAI) (43).	Full name Date of birth Age Contact information Occupation Last educational degree State of residency Ethnicity Ancestry Genealogy
Medical history	Personal and family history of cardiovascular, metabolic, endocrine, neoplastic, respiratory, psychiatric, neurologic, urinary, reproductive, gastrointestinal, rheumatologic, developmental, pregnancy, dermatologic, and ophthalmologic disorders, surgical history and medication use	Personal and family history of cardiovascular, metabolic, endocrine, neoplastic, respiratory, psychiatric, neurologic, urinary, reproductive, gastrointestinal, rheumatologic, developmental, pregnancy, dermatologic, and ophthalmologic disorders, surgical history, and medication use. Lupus-associated symptoms and clinical manifestations based on the Systemic Lupus Erythematosus Disease Activity Index (SLEDAI) (44) and the American College of Rheumatology (ACR) criteria (45) Treatment	Clinical site and neurologist where the participant is registered. Motor and non-motor symptoms Medication Specific scales: Unified Parkinson's Disease Rating Scale (MDS-UPDRS) (46) and the Hoehn & Yahr scale (47)
Mental health	State-Trait Depression Inventory (ST-DEP) (48) State-Trait Anxiety Inventory (STAI) (49) Paranoid ideation Psychoticism and others (50) Self-confidence Social competence Family support Ability of people to organize themselves in difficult times (51)	State-Trait Depression Inventory (ST-DEP) (48) State-Trait Anxiety Inventory (STAI) (49) Symptom Checklist, Revised (SCL-90-R) (52) Coping Strategies Inventory (CSI) (53) Mexican Resilience Scale (RESI-m) (51)	State-Trait Depression Inventory (ST-DEP) (48) State-Trait Anxiety Inventory (STAI) (49) Symptom Checklist, Revised (SCL-90-R) (52) Parkinson Anxiety Scale (54)
Cognitive function	Brief IQ test (Shipley-2) (55), Synchronization-continuation task (56) and Speech-to-Speech Synchronization task (57) Spatial n-back (0,2-b) task for assessing working memory during fMRI (58) Creyos’ computerized cognitive battery of 12 tasks (59)	Montreal Cognitive Assessment (MoCA) soon to be applied (60)	Montreal Cognitive Assessment (MoCA) (60) Creyos’ computerized cognitive battery, eight selected tasks (59)
Environmental factors	Parental Bonding Questionnaire (61) Childhood Trauma Questionnaire (62).		Questionnaires about pesticides, heavy metals, water, air or soil contaminants, exposure, and head trauma were also included.
Reproductive health	Menstruation Pregnancy Breastfeeding Use of hormonal treatments		PD women-specific questionnaire (63)
Lifestyle and quality of life	Pittsburgh Sleep Quality Index (64) World Health Organization Quality of Life (WHOQoL-bref) (65). 36-Item Short Form Health Survey (SF-36) (66)	Pittsburgh Sleep Quality Index (64) World Health Organization Quality of Life (WHOQoL-bref) (65). 36-Item Short Form Health Survey (SF-36) (66) Lupus Quality of Life (LupusQoL) (67)	Not applied.
Personality traits	Big Five Personality inventory (68) Behavioral Activation System and Behavioral Inhibition System (69)	Big Five Personality inventory (68)	Not applied.
Diet and eating behaviors	Food intake Food-related behaviors Traits suggestive of eating disorders (70)		Not applied.
Physical activity	Global Physical Activity Questionnaire (GPAQ) (71)		Not applied.
Substance use and abuse	Frequency and amount of: Tobacco (72), Alcohol Use Disorders Identification Test (AUDIT) (73), Cannabis Use Disorders Identification Test (CUDIT) (74), Consumption of other drugs Substance use disorder identification.	Not applied.	Frequency and amount of: Tobacco Alcohol Caffeine
Cognitive reserve	Not applied		The Cognitive Reserve Index Questionnaire (75)
Zygosity	Evaluation of the similitude of phenotypic physical traits and the frequency that their relatives confound twins. This questionnaire identifies if twins are identical (monozygotic) or nonidentical (dizygotic) with an accuracy of 98% (76)	Not applied.	
Laterality	Edinburgh Handedness Inventory (77)	Not applied.	

All three projects generally share an initial structure, with questionnaires focused on identifying socio-demographic traits and characterizing medical history and mental health. The shared technology information infrastructure of the registries has allowed us to design, implement, and validate surveys in one of the registries to further establish them in the other registries. In addition to these shared questionnaires, each registry includes surveys focused on bringing light on specific traits associated with the studied sample, such as zygosity in TwinsMX or cognitive impairment in MEX-PD.

Although the selection of each instrument responds to particular research questions, one primary guideline is the ability to produce harmonized measures to link and compare the data across registries and their counterparts in other populations (78). For instance, TwinsMX was built to be comparable to the Australian (79) and UK (80) Twins Registries. At the same time, MEX-PD is akin to the newly established Australian Parkinson's Genetics Study (81) and the Latin American Research Consortium on the Genetics of Parkinson's Disease (82).

### 2.3 Project identity

The term “-omics” encloses the acquisition, analysis, and interpretation of large datasets specific to various biological systems. These approaches, including genomics, proteomics, and metabolomics, allow for in-depth characterization of biological phenomena within specific contexts, thus helping to unravel the complexity of a given biological system or condition (83). In this context, the omics in our Consortium name, “MexOMICS” reflects our aim to generate and integrate omic data, particularly genomic and transcriptomic data, from Mexican individuals. Our goal is to offer a thorough comprehension of specific traits within our population by assessing clinical data and integrating the influences of genetic and environmental factors.

**Figure 1:**
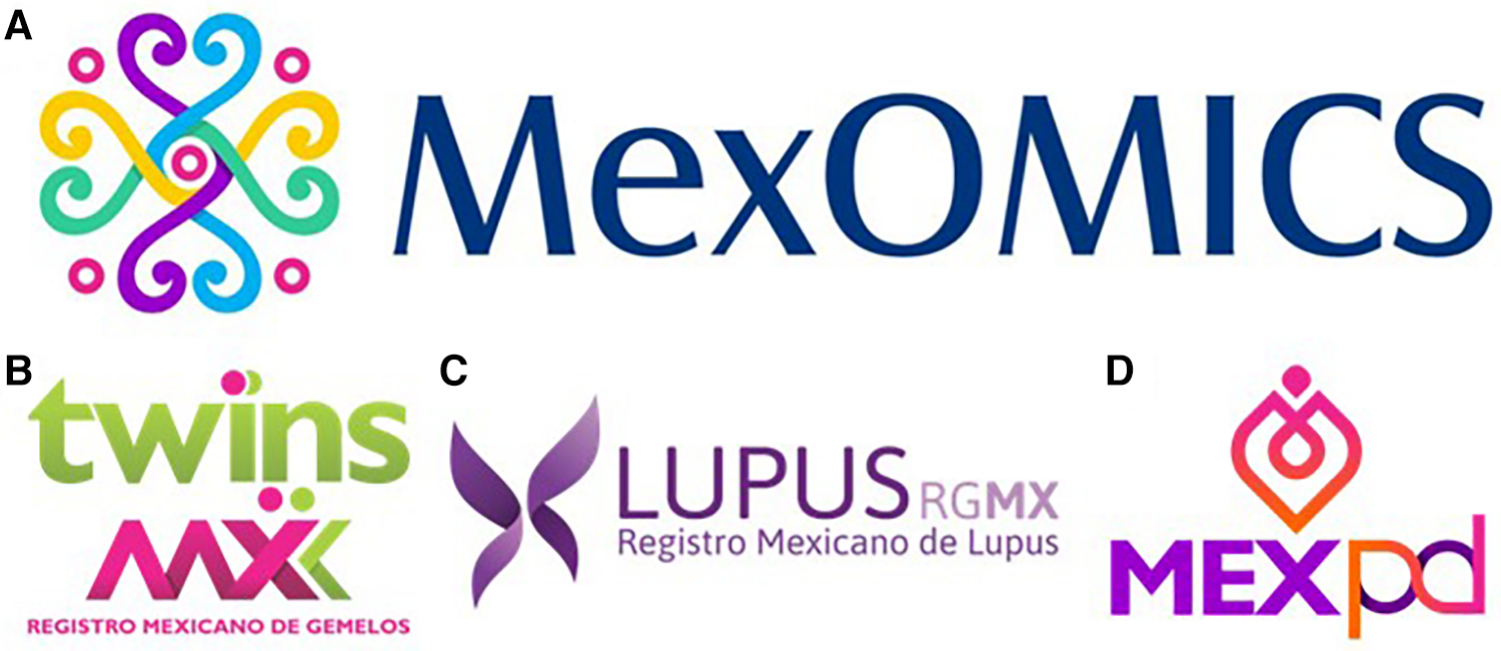
Logos designed for (**A**) MexOMICS, (**B**) twinsMX, (**C**) LupusRGMX and (**D**) MEX-PD.

The logo of “MexOMICS” is accompanied by four entangled DNA strands, each representing the conjunction of projects (TwinsMX, LupusRGMX, and MEX-PD) in a Mexican-styled design that resembles a prehispanic symbol known as ollin*,* which represents “movement” (Figure 1A).

For the Mexican Twin Registry (Registro Mexicano de Gemelos in Spanish), we selected the acronym TwinsMX; “Twins” to highlight the multiple births and “MX” for Mexican. The logo is centered in this acronym and includes “Registro Mexicano de Gemelos” in the bottom part. The “I” and “X” letters include two dots on the top, resembling a pair of heads, representing the pairs of twins (Figure 1B). Color palette includes bright green and a shade of pink known as Mexican pink in our culture; both colors are widely used on traditional artisanal crafts and therefore represent the vibrancy of our culture, as well as the spirit, charisma, liveliness, and vitality of our people. TwinsMX's website (https://twinsmxofficial.unam.mx/) includes the “Sign up” section, along with an “About us” section focused on the academic profiles of the TwinsMX team members, frequently asked questions, information about the projects well as an interactive section that allows the people to see how is the registry advancing through time. It also includes a “Blog” section that shares science-based information of interest in a non-technical language. TwinsMX was launched in May 2019 and represents the first platform implemented by our consortium.

For the Mexican Lupus Registry's acronym, the word Lupus was selected as the central element, followed by the letters “RG” from ReGistry and “MX” from MeXican. The registry's logo includes the acronym along with “Registro Mexicano de Lupus” in the bottom part and integrates a butterfly, which resembles the malar rash, one of the most common manifestations of SLE, along with a meaning of hope; the purple color palette was selected because of its broad utilization in awareness campaigns as it symbolizes a middle point between the intensity and motivation of the red and the calmness and stability of the blue (Figure 1C). The website (https://lupusrgmx.liigh.unam.mx/) was created considering the same color palette. It includes a “Who are we” section, which includes academic profiles of the research team members, a “Communication” part with the structure of an informative blog, a “Lives” section that shows the Facebook Live events hosted by members of the project, a “Contact” section to provide a communicative platform with the team, and the “Sign up” button to access to the registry. LupusRGMX was successfully launched in May 2021, was socialized through a live Facebook session, and has worked since then using these platforms.

The Mexican Parkinson's Research Network, Red Mexicana de Investigación en Párkinson in Spanish, is represented with the acronym “MEX-PD”, which includes “MEX” for Mexican and “PD” for Parkinson Disease (Figure 1D). The logo includes a tulip silhouette, a flower that has been broadly used to raise awareness about Parkinson Disease since a horticulturist living with PD developed a new variant of tulip and named it after Dr. James Parkinson, the first physician to describe and document the disease. MEX-PD website (https://mexpd.liigh.unam.mx/) includes a “Frequently asked questions” section, an “About us” section that includes the academic information about the team, and a “Pre-registry” option. MEX-PD was launched in July 2021.

### 2.4 Inclusion and exclusion criteria

For the TwinsMX cohort, Mexican multiple-birth individuals of all ages are welcome to participate in the online survey. Minors must obtain consent from a parent or legal guardian and are provided with age-appropriate questionnaires for minors, parents can assist pre-pubescent children in completing them. For the MRI study, only twin participants between 18 and 60 years of age with no major neurological disease (e.g., moderate or severe traumatic brain injury, neurodegenerative disease, tumor, stroke, and epilepsy), intellectual disability (an estimated IQ below 70), and who can read are included.

In LupusRGMX, Mexican individuals aged over 18 years with SLE diagnosis were included. SLE participants must have a confirmed diagnosis and fulfill ≥4 American College of Rheumatology (ACR) criteria (Hochberg) at the moment of diagnosis (45). Current or past corticoids and immunosuppressants usage were also used to confirm SLE diagnosis. Disease activity, additional treatments, and comorbidities were not considered as exclusion criteria.

MEX-PD participants were required to have been born in Mexico and/or have spent most of their lives in the country and must be at least 45 years old. PD diagnosis must be confirmed by neurologists specialized in movement disorders, based on the UK Brain Bank Criteria.

LupusRGMX and MEX-PD included non-affected controls who fulfilled the same age criteria and were not consanguineous with participants with SLE and PD, respectively. Additionally, people with another autoimmune disease were excluded from the LupusRGMX control cohort, whereas individuals with a diagnosis of neurodegenerative diseases were excluded from the MEX-PD control cohort.

### 2.5 Volunteers recruitment

As one of the main objectives of the registries is to include as many Mexicans as possible across the country, it became evident that a decentralized approach was needed. Given the varied nature of each registry, specific procedures for participant recruitment have been followed: for TwinsMX, the enrollment is made through social media campaigns and on-site event invitations. Given the complexity of PD diagnosis, the recruitment in MEX-PD requires the clinical expertise of neurologists; a team of neurologists from different sites across Mexico has been assembled, and volunteers are being identified and registered through their practice. For LupusRGMX, the recruitment relies on a hybrid strategy: A team of rheumatologists actively register patients in their practice through social media and on-site events where volunteers are invited to self-register.

### 2.6 Genetic data acquisition

The collection of biological samples through buccal swabs for DNA acquisition is being performed at multiple sites for the three cohorts, allowing for the representation of diverse genetic backgrounds. Additionally, in LupusRGMX, DNA has also been isolated from blood samples.

For TwinsMX, genotypes were generated using the Illumina Infinium Global Diversity Array-8 v1.0 array, which comprises a 1,825,277 SNPs global backbone designed for cross-population imputation coverage of the genome [https://www.illumina.com/products].

In LupusRGMX, whole genome sequencing was performed by Novogene Inc. in Sacramento, CA, United States (https://www.novogene.com/). The genome libraries were prepared (350 bp), and sequencing was performed using the Illumina NovaSeq X Plus Series® platform with a paired-end 150 bp sequencing strategy, with 90G raw data per sample and a coverage depth of 4X. Simultaneously, 50 samples have been collected to perform single-cell RNA-seq of peripheral blood mononuclear cells.

For MEX-PD genotyping is being performed through the support and collaboration with the Global Parkinson's Genetics Program (GP2) (84) and the Latin American Research Consortium on the Genetics of Parkinson's Disease (LARGE-PD) (82). The genotyping platform is the Illumina NeuroBooster array (NBA), containing 1,914,934 variants and specifically designed to screen for genetic variation in neurological disorders across diverse populations (85).

## 3 Results

### 3.1 Progress at a glance

As of June 2023, our registries included 2,915 volunteers for TwinsMX (April 28th, 2023), 1,761 participants for LupusRGMX (June 21st, 2023), and 750 for MEX-PD (June 29th, 2023) (Table 2). Additionally, for LupusRGMX, 153 participants were registered as controls, whereas MEX-PD had 397 volunteers registered as non-affected controls. Female participation represents 71.9% of TwinsMX, 94.3% in LupusRGMX, and 59.2% in MEX-PD, whereas 28.1%, 6.7% and 40.8% of participants were male in each study.

**Table 2:** Sociodemographic characteristics of volunteers across registries.

Information	TwinsMX (*n* = 2,915*)	LupusRGMX (*n* = 1,761)	MEX-PD (*n* = 750)
Gender (*n*, %)			
Female	2,096 (71.9%)	1,662 (94.3%)	444 (59.2%)
Male	819 (28.1%)	99 (6.7%)	306 (40.8%)
Age at enrollment (mean, SD)	29.2 ± 11.2	37.1 ± 10.9	62.17 ± 10.6
Time with the diagnosis (mean, SD)	NA	8.5 ± 7.8	6.7 ± 5.7
Age at diagnosis (mean, SD)	NA	28.3 ± 10.9	59.7 ± 11.8
Genetic samples collected (*n*, %)	232 (7.9%)*	108 (6.1%)	495 (66%)
MRI sampled (*n*)	267 (9.1%)	To start in Q2 2024	To start in Q2 2024
Cognitive function evaluations	267 (9.1%)**	To start in Q2 2024	376 (50.1%)

* Individual subjects.

** Subjects in MRI study.

**Figure 2:**
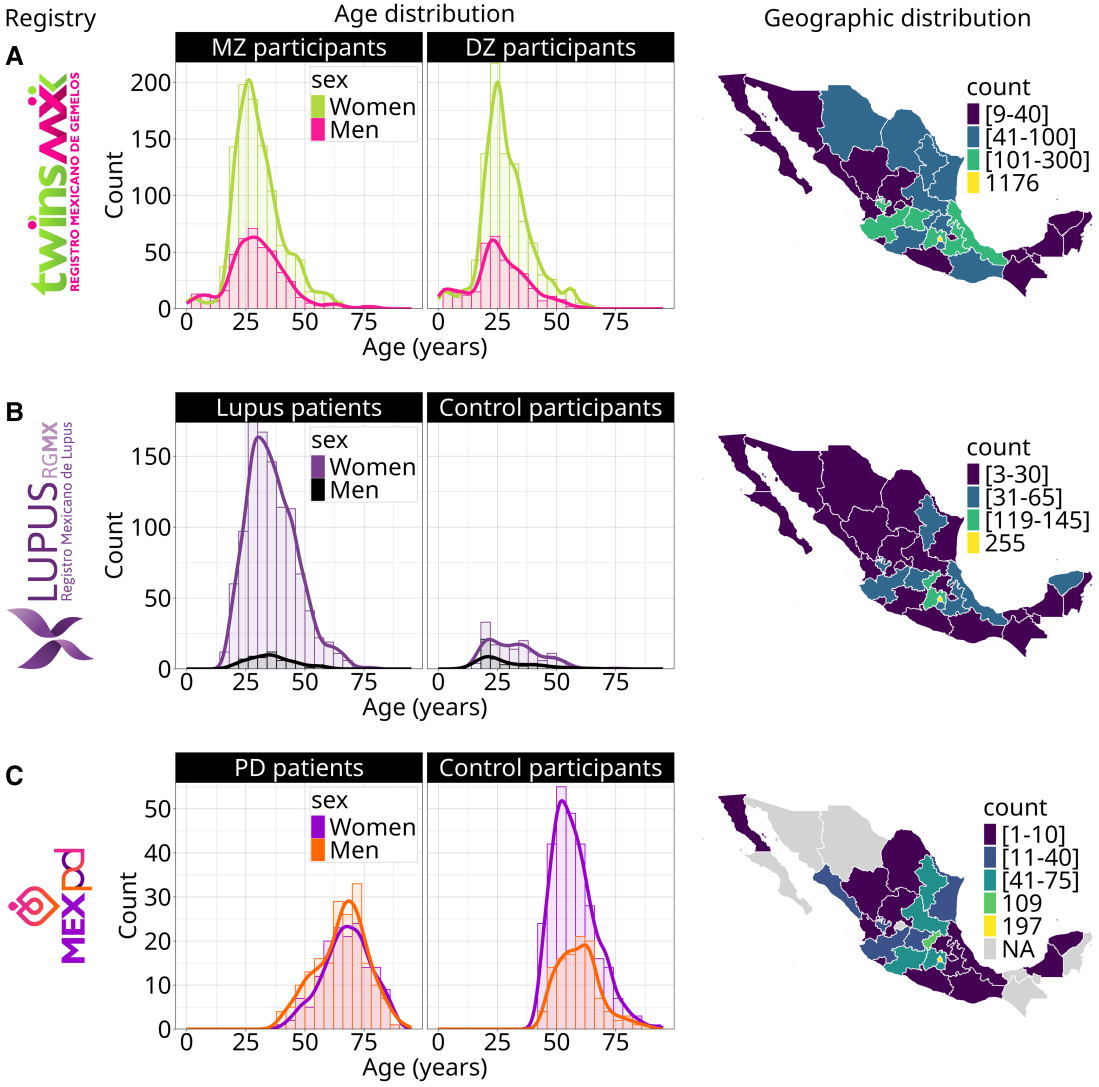
Geographic and age distribution of volunteers across registries. Age distribution for a = TwinsMX, b = LupusRGMX (*n* = 1,761) and c = MEX-PD (*n* = 774). Geographic distribution for a = TwinsMX, b = LupusRGMX (*n* = 1,761), c = MEX-PD (*n* = 750).

TwinsMX covers an age range from 0 to 80 years, with the majority between 20 and 40 y.o. (Figure 2A), and a mean age of 29.2 ± 11.2 years at the moment of enrollment. Age distribution among monozygotic and dizygotic participants is shown in Figure 2, and a similar pattern can be observed in both groups.

In the case of LupusRGMX, the age range is between 18 and 79 years (Figure 2B), with the majority of the cohort aged between 20 and 45 and a mean age of diagnosis of 28.3 ± 10.9 years. Healthy controls' age distribution is also exhibited, with an age range from 10 to 60 years and the majority of volunteers being in their early 20s.

For MEX-PD, participants' ages range from 40 to 90 years (Figure 2C), with the majority being between 60 and 80 and a mean age of 62.17 ± 10.6 years. Control participants exhibited a similar age range, with the majority being between 40 and 60 y.o.

As mentioned, genetic analyses and other clinical measurements can help enrich the information collected through the registries, giving insights into the biological conditions of our participants. In this line, our team has been recovering DNA samples from volunteers of the three projects. As of June 2023, TwinsMX had collected 232 swab samples, of which 47 have already been genotyped. In LupusRGMX 109 samples have been recovered to perform whole genome sequences, of which 71 have been successfully sequenced: 61 from volunteers with SLE and ten healthy controls. In the case of MEX-PD, 495 samples have been obtained to perform whole genome genotyping.

As the nervous system is compromised in Parkinson's Disease, cognitive functions are evaluated with the Montreal Cognitive Assessment (MoCA). As of June of 2023, 126 controls and 250 patients have been evaluated. Due to the high prevalence and severity of neurologically related manifestations in SLE (also known as neuro-lupus), LupusRGMX will apply 90 MoCA evaluations by 2024. Additionally, structural, functional, and spectroscopy brain images have been acquired through MRI sessions of a subset of 267 TwinsMX volunteers (June 2023), although 500 participants are expected to be collected by the Spring of 2024. MEX-PD and LupusRGMX will perform at least 90 MRI evaluations each by 2024.

Regarding the geographical coverage of the three projects, we have a substantial number of volunteers from the center of Mexico, probably due to the location of the leading laboratories (Mexico City and Queretaro). In TwinsMX (Figure 2A), the majority of registers are concentrated in Mexico City (*n* = 1,176), Mexico State (*n* = 295), Veracruz (*n* = 149), Jalisco (*n* = 111), Puebla (*n* = 105), and Guanajuato (*n* = 101); with less participation among people from the north-western and south regions. For LupusRGMX (Figure 2B), we can observe higher participation among people living in Mexico City (*n* = 255), Mexico State (*n* = 145), and Queretaro (*n* = 119), whereas the west and northern west regions are the least represented. In the case of MEX-PD (Figure 2C) most of the participants come from Mexico City (*n* = 197), Queretaro (*n* = 109), Morelos (*n* = 75), Michoacan (*n* = 72), San Luis Potosí (*n* = 71), Mexico State (*n* = 62), and Nuevo Leon (*n* = 42); the states with no volunteers registered are shown in light gray.

### 3.2 Challenges and limitations

Implementing a National Registry always faces challenges, especially at the beginning. For instance, TwinsMX relies entirely on the self-registration of volunteers who are reached by our invitations through social media or events since there is no database available to allow us to identify and contact potential participants, which limits the scope of the project. Besides, it is necessary that all the siblings of multiple births complete the surveys. Still, in most cases, only one of the twins is interested or motivated to finish the questionnaires, which leads to incomplete data. Another limitation is that in Mexico, there is a word used explicitly for fraternal twins, “cuate” (/'kwate/), thus often, they do not identify themselves as twins, making them think that they are excluded from the registry. More vigorous and targeted media campaigns are being implemented to address this issue.

LupusRGMX mainly works on a voluntary self-registration basis of people reached by our communication campaigns. By far, the main obstacle we have faced has been reaching Mexican individuals with SLE with the interest and disposition to start and complete the surveys: this is reflected in the fact that from the 1761 started enrollments, only 1,271 have completed the clinical questionnaire. Different strategies have been undertaken to increase survey completion, such as spacing out survey invitations and motivating participants through group dynamics, contests, and offering giveaways. In addition, since the beginning, LupusRGMX has tried to approach rheumatologists through the Mexican College of Rheumatology to establish collaborations in which the rheumatologist gives a formal clinical evaluation at the beginning of the registry and helps their patients through the initial clinical manifestations questionnaires, improving the reliability of our data. As of June 2023, only 147 participants have been registered through this option. With the knowledge that self-reported data can be threatened by self-reporting bias and thus considered unreliable (86), strategies are being undertaken to assess them; we are currently working on comparing the outcomes from self-reported instruments with those provided by patients' medical practitioners to evaluate reliability.

Similarly, as MEX-PD patients must be registered through a Neurologist with expertise to perform a clinical evaluation, reaching people with access to this level of medical care has been challenging. The relative shortage of Neurologists with valid certification in Mexico (87), along with their substantial workloads, driven by the necessity to attend to a large number of patients, limits the scope of the registry. Nonetheless, 774 participants have been enrolled so far. Furthermore, as aforementioned, Neurologists in Mexico usually have a heavy workload, making the survey's completion daunting. To ease this task, a trained assistant team helps patients complete particular surveys (i.e., environmental exposure). Moreover, we are currently working on transferring suitable instruments for participants to self-complete.

Likewise, a shared challenge between the registries is acquiring data from more diverse individuals within Mexico. Reviewing the current progress of the cohorts, it became evident that most enrollments are representative of the central states of Mexico and from specific regions where we have collaborating associations. For this reason, we are working on specifically targeting the underrepresented regions of the country, taking advantage of online tools, using social media, and establishing new collaborations. However, Magnetic Resonance Imaging, performed in suitable TwinsMX participants and blood samples for LupusRGMX, demands on-site presence of participants and is only carried out in Queretaro, limiting the participation of people from northern or southern states in the country. These techniques' complexity and required infrastructure prevent its mobility to other Mexican states.

Finally, because national registries are rare in Mexico, constant invitation reminders via traditional and social media are required to boost participation continuously. As of 2020, 72.0% aged six or older in Mexico residents are internet users (88). Therefore recruitment through social media has shown to be a very effective tool, with similar models successfully implemented in other registries (89, 90). As of June 2023, our registries have implemented an integrative approach through social media, turning them into reliable platforms for the exposition and discussion of topics associated with each community.

### 3.3 Sharing is caring: community building

In Mexico, according to the General Science, Technology and Innovation State Inform 2018, over 69.8% of the population perceive that the knowledge generated through scientific and technological research positively impacts our economy, and up to 84.5% think that scientific research plays a vital role in the technological development of the country. Still, 45.7% of the population perceive the scientific community as dangerous due to their knowledge and the potential misuse of their discoveries by third parties (91). In this sense, as a consortium, we believe that the participants in the three registries are not subjects of study but active citizens with a major role in biomedical research. As such, it is imperative to provide informed consent, maintain transparent procedures, disclose progress reports, and open communication channels where participants can provide feedback.

As twins, Lupus, and Parkinson's communities in Mexico were already active, one of the first steps in building the three registries was approaching community leaders, civil organizations, and foundations, which have been critical in reaching participants and gaining credibility in their communities. TwinsMX, the first of this consortium, received support from the Multiple Birth Association (Asociación de Nacimientos Múltiples, A.C.). TwinsMX now holds a significant community, with a substantial presence in social media with over 3.2 K followers on Facebook (https://www.facebook.com/TwinsMXofficial) and 748 on Instagram (https://www.instagram.com/twinsmxofficial). The TwinsMX's team constantly works on generating informative content (https://twinsmxofficial.unam.mx/blog/) and fosters participation in community activities.

Furthermore, LupusRGMX receives direct support and active participation from representatives of the biggest communities of people with Lupus in the country: LUPUS MX (https://www.facebook.com/LUPUSMXOficial), Fundación Proayuda Lupus Morelos A.C. (https://www.facebook.com/LUPUSMORELOS), the Centro de Estudios Transdisciplinarios Athié-Calleja por los Derechos de las Personas con Lupus A.C. (https://www.facebook.com/Cetlu), and Despertar de la Mariposa A.C. (https://www.facebook.com/DespertarLupus); who have reviewed the surveys, and expressed their opinions and needs. We have also approached rheumatologists and the Colegio Mexicano de Reumatología to establish collaborations to help provide a more integrated evaluation of SLE in our cohort. The formation of this community has helped us identify necessities and areas of opportunity for clinicians and researchers to generate knowledge with future applications and impact on the lives of people with lupus. Through contact with experts on topics of interest such as pediatric SLE, grief associated with a chronic disease diagnosis, and reproductive health, we have established monthly open talks through Facebook Live sessions (https://www.facebook.com/lupusrgmx). Besides these talks, the team of LupusRGMX has been working on providing other sources of reliable information (https://lupusrgmx.liigh.unam.mx/comunicacion.html) and actively participating in activities for lupus awareness in Mexico.

Similarly, MEX-PD has sought support from the Red Mexicana de Asociaciones de Párkinson (https://www.facebook.com/RedMXdeAsociacionesdeParkinson) and different communities of people with Parkinson's and their loved ones, including Parkinson Laredo (https://www.facebook.com/groups/768321520680203) and Asociación Mexiquense de Parkinson IAP (https://www.facebook.com/parkinsonmexiquense).

### 3.4 Key findings from ongoing research

Deeper analyses of the sociodemographic and clinical characteristics of each registry will help answer each project's specific questions; these analyses are and will continue to be presented in independent articles. However, we summarize here the main findings to exemplify the potential of these resources.

The TwinsMX registry aims to understand the interplay of genetic and environmental factors influencing the most prevalent diseases, subclinical symptoms, and cognitive aspects of the Mexican population. The first steps of this project have been previously described (37). The latest results include a study on myopia and astigmatism, revealing notably high heritability (over 80%) estimated for both conditions, accompanied by a substantial cross-trait genetic correlation (92). These findings are consistent with prior research conducted in other populations, suggesting that both traits are influenced by a shared set of genes. One of the further goals of this project is to explore associations of genetic variants with other publicly relevant health conditions, as well as neuropsychiatric measures such as depression and anxiety symptoms. For instance, our latest published advance for this project (92) revealed a high prevalence of gastritis, colitis, overweight, allergic rhinitis, and gastroesophageal reflux, in addition to myopia and astigmatism. On the other hand, anxiety, depression, insomnia, migraine, attentional deficit/hyperactivity disorder, and obsessive-compulsive disorder were the mental health conditions with the highest occurrence. Additionally, we have reported our progress in collecting MRI data, including high resolution brain structural images, resting state functional MRI, magnetic resonance spectroscopy and task-related functional MRI (40). Altogether, this project is expected to aid on the characterization of the health profiles of our population, and the relative influence of genetic and environmental factors on a great variety of health, cognitive and brain phenotypes in the Mexican population.

Hernandez-Ledesma et al. (38) used LupusRGMX data to evaluate quality of life disparities, finding a lower quality of life in people with lupus than healthy participants, and that the quality of life was predicted by socioeconomic status, delay in diagnosis, and corticosteroid consumption, highlighting that lower socioeconomic status leads to a lower quality of life. Pedraza-Meza et al. (93) analyzed how social, clinical, psychological, and demographic variables affect social and temporal decision-making in people with lupus, reporting the importance of age and hostility to predict social decisions, as well as anxiety and obsessive-compulsiveness to predict temporal decisions, whereas clinical factors, i.e., being in remission and taking glucocorticoids, predict both kinds of decisions.

Regarding MEX-PD, Lázaro-Figueroa et al. (39) described a general analysis of the cohort demographic and clinical data. The age of onset of Parkinson's Disease (PD) was found to be younger than the reported for other populations as 19.8% of patients with PD were identified as early onset (age <50 years). Contrary to what was previously reported in other populations, the MEX-PD cohort did not show an association between head injury and PD. The more frequent initial motor symptom that MEX-PD patients reported was tremor in the upper limbs, and the more frequent treatment was levodopa. These preliminary results give new information about the characteristics of PD in the Mexican population.

### 3.5 Perspectives

From a general perspective, TwinsMX, LupusRGMX, and MEX-PD will allow us to characterize the genetic and environmental contribution of different traits and diseases with data collected in the Mexican population, which is heavily underrepresented in epidemiological and genetic studies.

Projects such as the MXB, the MAIS, and the MCPS studies represent a breakthrough in the knowledge of genetic diversity in Mexico and have added invaluable data regarding the genetic structure of the Mexican population. However, there remains an unmet need to expand our understanding of the impact of genetic variants, environmental factors, and their interactions on health-related traits. Overall, this characterization will provide medically relevant information to design, execute, and monitor clinical trials, and improve the time and accuracy of diagnosis, treatments, and therapies.

The collaborative design implemented in MexOMICS' registries, which contemplates recontacting based on email and integration of new questionnaires, alongside genetic data acquisition, facilitates the conduct of longitudinal studies. Furthermore, the shared questionnaires and variables recorded among the three registries enables data comparison between studies (38).

On the other hand, the registries within MexOMICS are also expanding to include non-invasive techniques to visualize and analyze brain structure and function. Multimodal neuroimaging will aid in the characterization of brain-health associations and their variability associated to genes and environment in our cohorts.

In particular, for TwinsMX, we expect to start to collect DNA from direct relatives of twins individuals (i.e., parents and not-twins siblings) to build a biobank. In the future, it is expected that we will acquire data through electroencephalography (EEG) to further investigate associations with cognitive performance. Meanwhile, for LupusRGMX MRI and cognitive function evaluations will be performed in at least 90 participants to explore the underlying functional and structural brain substrate. Finally, MEX-PD will also acquire imaging data through MRI, employing state-of-the-art techniques such as resting-state functional imaging, high-resolution multicontrast structural acquisitions, diffusion, and neuromelanin-weighted imaging. By also exploring non-affected controls, the main aim will be to characterize the functional and structural brain substrate of the clinical and cognitive deficits identified in our cohort.

## 4 Conclusions

Patient registries are fundamental tools in public health research, providing comprehensive population-based data on diseases' etiology and progression. Establishing registries in Mexico, including those initiated at the Universidad Nacional Autónoma de México through the MexOMICS Consortium, has enriched the health research landscape with epidemiological and genetic information. These registries enable researchers to explore the complex interplay between genetic factors and environmental influences in traits in the general population by studying twin participants and those involved in complex diseases like systemic lupus erythematosus and Parkinson's disease. However, limitations, such as inconsistent data entry and patient participation, together with potential biases due to voluntary participation, must be addressed to ensure that the creation of these databases can serve as robust foundations for subsequent studies and investigations. Future directions involve refinement and expansion, optimizing experimental design, data collection methodologies, and fostering interdisciplinary collaboration.

The MexOMICS Consortium's registries represent a valuable resource for addressing questions associated with health and disease in the Mexican people.

## Data Availability

The raw data supporting the conclusions of this article will be made available by the authors, without undue reservation.
